# Topical Epidermal Growth Factor in Acne Vulgaris and Acne‐Related Sequelae: Mechanistic Rationale, Clinical Evidence, and Formulation Challenges

**DOI:** 10.1111/jocd.71189

**Published:** 2026-09-11

**Authors:** Nasim Gholizadeh, Ghasem Rahmatpour Rokni, Maryam Sahafi, Omid Mohaghegh, Ardalan Etemadzadeh, Reyhaneh Pasandipour, Hamed Fakhrabadi, Ghazal Zoghi

**Affiliations:** ^1^ Department of Dermatology, Faculty of Medicine Mazandaran University of Medical Sciences Sari Iran; ^2^ Student Research Committee, Faculty of Medicine Hormozgan University of Medical Sciences Bandar Abbas Iran; ^3^ Student Research Committee, School of Medicine Iran University of Medical Sciences Tehran Iran; ^4^ Endocrinology and Metabolism Research Center Hormozgan University of Medical Sciences Bandar Abbas Iran

**Keywords:** acne scars, acne vulgaris, combination therapy, epidermal growth factor, inflammation, review, tissue repair, topical treatment, wound healing

## Abstract

**Background:**

Acne vulgaris (AV) is a common chronic inflammatory skin disorder, particularly among adolescents and young adults, and its management remains limited by variable treatment response, poor adherence, and treatment‐related adverse effects.

**Aims:**

This review critically evaluates the mechanistic rationale, clinical evidence, safety considerations, and formulation challenges relating to topical epidermal growth factor (EGF) in active acne vulgaris and acne‐related sequelae.

**Methods:**

PubMed was searched from database inception through June 30, 2026, using predefined MeSH and free‐text terms related to AV, acne scarring, epidermal growth factor, EGF receptor (EGFR) signaling, keratinocytes, sebocytes, inflammation, skin‐barrier repair, wound healing, and topical delivery. PubMed‐indexed articles were selected through title‐and‐abstract screening followed by full‐text assessment. Human acne‐related studies were prioritized, while relevant preclinical studies were included to evaluate mechanistic, safety, and formulation‐related evidence. Owing to substantial heterogeneity in study design and indication, evidence was synthesized narratively rather than quantitatively.

**Results:**

Preclinical studies suggest that EGF/EGFR signaling may influence keratinocyte inflammation, migration, proliferation, and tissue repair; however, most of this evidence derives from non‐acne cellular or wound‐healing models. Direct clinical evidence for active acne is limited to two small, short‐term split‐face studies that reported preliminary within‐study improvements in lesion‐related outcomes. Separate small studies have evaluated topical EGF‐containing formulations for established atrophic acne scars or recombinant human EGF (rhEGF) as an adjunct to fractional laser treatment. These studies do not demonstrate prevention of new acne scars, and evidence from scar or post‐procedural settings cannot be extrapolated to active acne treatment. Heterogeneity in study design, formulation, indication, outcome assessment, and follow‐up prevents firm conclusions regarding clinically meaningful efficacy, comparative effectiveness, or long‐term safety. The single‐database, English‐language narrative‐review methodology also limits the comprehensiveness and certainty of the synthesis.

**Conclusions:**

Available studies provide preliminary biological rationale and limited clinical signals for topical EGF in acne‐related care, but the evidence is insufficient to establish clinically meaningful efficacy or an evidence‐based therapeutic role in active acne vulgaris. Evidence for topical EGF serum used alone on established atrophic scars remains weak, whereas the comparatively broader evidence concerns rhEGF as an adjunct after fractional laser treatment for established scars. Even in this procedural setting, heterogeneous protocols and limited evidence certainty preclude routine recommendation. Topical EGF should therefore remain investigational, and larger randomized controlled trials using standardized formulations, active comparators, indication‐specific outcomes, and longer safety follow‐up are required.

## 1 Introduction

Acne vulgaris (AV) is a chronic inflammatory disease of the pilosebaceous unit that predominantly affects adolescents and young adults, although it may persist into adulthood. It represents one of the most common inflammatory dermatoses worldwide [1]. The pathogenesis of acne involves four interrelated processes: increased sebum production, abnormal follicular keratinization, dysbiosis involving acne‐associated *Cutibacterium acnes* strains, and inflammatory responses [2, 3, 4].

Despite numerous available therapeutic options, significant treatment challenges persist in acne management. Current first‐line treatments include topical retinoids, benzoyl peroxide, topical antibiotics, and their combinations [5, 6]. However, limitations related to efficacy, tolerability, adherence, and antimicrobial resistance support continued investigation of additional treatment approaches. Treatment limitations include incomplete efficacy, undertreatment, and poor adherence to existing regimens [7]. The prolonged duration of acne treatment, often extending for months or years, may contribute to poor adherence. In addition, adverse effects such as irritation, dryness, and photosensitivity may further reduce adherence [7]. Meanwhile, antibiotic resistance represents a growing concern, with widespread and prolonged antibiotic use contributing to the emergence of resistant *C. acnes* strains [8, 9].

Epidermal growth factor (EGF) is a 53‐amino acid single‐chain polypeptide that exerts its effects through binding to the EGF receptor (EGFR) [10]. EGF/EGFR signaling participates in cellular processes including survival, proliferation, migration, differentiation, and tissue repair [11]. In skin models, these effects include modulation of keratinocyte proliferation, migration, re‐epithelialization, and epidermal homeostasis; however, their direction and magnitude depend on cell type, exposure conditions, and the experimental context [12, 13, 14, 15, 16, 17, 18, 19, 20].

This narrative review critically evaluates the mechanistic rationale, clinical evidence, safety considerations, and formulation challenges concerning topical EGF in acne vulgaris and acne‐related sequelae. Evidence relating to active inflammatory and non‐inflammatory acne lesions is considered separately from evidence concerning atrophic acne scars, procedural adjunctive therapy, and post‐procedural skin repair. Preclinical wound‐healing and formulation studies are discussed as indirect mechanistic evidence and are not considered proof of clinical efficacy in active acne. The review also evaluates whether the available evidence supports a defined clinical position for topical EGF in active acne, scar prevention, treatment of established atrophic scars, or post‐procedural care.

## 2 Methods

### 2.1 Review Design

This study was conducted as a structured narrative review. Its purpose was to critically integrate heterogeneous clinical, mechanistic, safety, and formulation evidence concerning topical EGF in active acne and acne‐related sequelae. The review was not prospectively registered, and it was not designed as a systematic review or meta‐analysis.

### 2.2 Search Strategy

A structured literature search was conducted in PubMed from database inception through June 30, 2026. The final search was executed on July 2, 2026, and June 30, 2026 was used as the prespecified publication‐indexing cutoff. Medical Subject Headings (MeSH) and free‐text terms were combined to identify publications related to AV, acne scarring, epidermal growth factor, recombinant human epidermal growth factor, epidermal growth factor receptor signaling, keratinocytes, sebocytes, inflammation, skin‐barrier function, wound healing, and topical delivery systems.

The principal search strategy combined terms related to AV or acne scarring with terms related to epidermal growth factor or recombinant human epidermal growth factor. Additional targeted searches were conducted to identify studies addressing acne‐relevant mechanisms and topical EGF formulation or delivery. The complete PubMed search strategies are provided in Table S1.

### 2.3 Eligibility Criteria

Peer‐reviewed English‐language articles indexed in PubMed were eligible when they addressed one or more of the following areas: topical EGF in active AV; EGF in acne‐scar prevention or treatment; EGF as an adjunct to acne‐related procedural interventions; EGF or EGFR signaling in acne‐relevant keratinocyte or sebocyte models; acne‐associated inflammation or skin‐barrier dysfunction; wound healing relevant to acne sequelae; or topical EGF formulation and delivery.

Human clinical studies directly evaluating active acne lesions, acne scars, or acne‐related procedures were prioritized. Preclinical studies and studies conducted in non‐acne wound‐healing models were included only when they provided mechanistic, safety, or formulation evidence relevant to the proposed topical use of EGF. The indirect nature of such evidence was considered when interpreting the findings.

Articles were excluded if they were not indexed in PubMed, were not sufficiently relevant to EGF or EGFR biology and acne‐related outcomes, were duplicate reports, were retracted, or did not provide sufficient methodological information for interpretation.

### 2.4 Study Selection and Evidence Synthesis

Study selection was conducted in two stages. Titles and abstracts were initially screened for relevance, followed by full‐text assessment of potentially eligible publications. Two reviewers independently conducted title‐and‐abstract screening and full‐text assessment using the predefined eligibility criteria. Disagreements were resolved through discussion and consensus, with consultation of an additional author where necessary. The included evidence was categorized as relating to active acne treatment, acne‐scar prevention or treatment, procedural adjunctive therapy, preclinical mechanisms, safety, or formulation and delivery.

### 2.5 Critical Appraisal and Synthesis

Because this was a narrative review encompassing clinical trials, uncontrolled pilot studies, retrospective clinical data, in vitro experiments, animal wound models, formulation studies, and systematic‐review evidence, a single formal risk‐of‐bias instrument was not applied across all included publications. Clinical studies were instead appraised descriptively according to design, sample size, control group, blinding, follow‐up, outcome assessment, and applicability to the stated clinical indication.

Quantitative synthesis was not undertaken because the evidence was limited and substantially heterogeneous with respect to indication, study population, experimental model, EGF formulation, comparator, treatment duration, and outcome measure. Findings were therefore synthesized narratively and interpreted according to their directness and methodological limitations.

## 3 Pathogenesis of Acne Vulgaris

### 3.1 Overview of Key Mechanisms

AV arises from four interlocking pathogenic mechanisms—sebum overproduction, follicular hyperkeratinization, *C. acnes* colonization, and an escalating inflammatory cascade. EGF/EGFR signaling has been linked experimentally to some of these pathways; however, its relevance to acne treatment remains incompletely defined and is based largely on indirect mechanistic evidence. Table 1 offers a concise mechanistic overview of AV pathogenesis.

**TABLE 1:** Concise mechanistic overview of AV pathogenesis.

Pathogenic step	Key molecular/physiological drivers	Representative recent evidence	Hypothesized relevance of EGF/EGFR signaling
Sebum overproduction	Androgen/IGF‐1–mTORC1 axis Altered lipidomics (↑triglycerides, ceramides) Diet‐induced SREBP‐1c up‐regulation	Quantitative lipidomics demonstrated altered skin‐surface lipid profiles in patients with acne, including increased levels of several triglyceride and ceramide classes, together with evidence of impaired skin‐barrier function [21]	Direct sebosuppressive activity of EGF has not been established. In SZ95 sebocytes, EGF stimulated proliferation and IL‐6 secretion and, when combined with palmitic acid, reduced lipid accumulation while enhancing hyperproliferative and inflammatory transcriptional responses. A small split‐face clinical trial reported reduced surface‐sebum output, but this finding has not been independently replicated and does not establish a direct sebocyte mechanism [22, 23]
Follicular hyperkeratinization	Dysregulated keratinocyte differentiation ↑IL‐1α stimulates comedogenesis Abnormal corneocyte cohesion	Abnormal follicular keratinization and microcomedone formation are central early events in acne pathogenesis [2, 3]	EGF promotes keratinocyte proliferation and migration in experimental wound‐healing models. These effects could theoretically support lesion repair, but because acne involves abnormal follicular proliferation and differentiation, excessive or prolonged EGFR stimulation could also aggravate hyperkeratinization. The net effect of topical EGF on microcomedone formation remains unknown [2, 3, 12, 15, 18, 19]
*C. acnes* colonization	Lipid‐rich microenvironment favors anaerobic growth Biofilm formation enhances virulence	A 2025 integrated metagenomic and lipidomic study identified reduced microbial diversity and acne‐associated lipid alterations in moderate acne. Notably, the abundance of *C. acnes* itself was not increased, indicating that community composition and microbial function may be more relevant than total bacterial abundance [24]; comprehensive immunological review confirms microbiome's central role [25]	In cultured human keratinocytes exposed to C. acnes, rhEGF reduced IL‐1α, IL‐8, and TNF‐α expression and decreased TLR2 expression and NF‐κB activation. The study did not evaluate topical delivery, bacterial viability, follicular colonization, or clinical acne outcomes [26]
Inflammatory cascade	TLR2/NLRP3 activation Th17 cytokines (IL‐17, IL‐23) Oxidative stress and lipid peroxidation	Genome‐wide analyses reveal broad up‐regulation of chemokines and MHC‐II genes in lesional skin [25]; lipid peroxidation amplifies inflammasome activation [27]	EGF reduced a substantial subset of IFN‐γ‐induced inflammatory transcripts in cultured keratinocytes; however, this was a non‐acne inflammatory model and its clinical relevance to topical treatment remains uncertain [28]

Abbreviations: AV, acne vulgaris; *C. acnes*, *Cutibacterium acnes*; EGF, epidermal growth factor; IFN‐γ, interferon gamma; IGF‐1, insulin‐like growth factor‐1; IL, interleukin; MHC‐II, major histocompatibility complex‐II; mTORC1, mammalian target of rapamycin complex 1; NLRP3, nucleotide‐binding oligomerization domain‐like receptor pyrin domain containing 3; rhEGF, recombinant human epidermal growth factor; SREBP‐1c, sterol regulatory element binding protein 1c; Th17, T helper 17; TLR2, toll‐like receptor 2; TNF‐α, tumor necrosis factor alpha.

### 3.2 Potential Mechanistic Relevance of EGF/EGFR Signaling

Experimental evidence indicates that EGF/EGFR signaling can modify inflammatory and migratory responses in keratinocytes, but the findings are model‐dependent. In cultured keratinocytes exposed to *C. acnes*, recombinant human EGF (rhEGF) reduced IL‐1α, IL‐8, and TNF‐α expression and decreased TLR2/NF‐κB signaling; however, this study evaluated cytokine responses rather than bacterial viability or clinical acne lesions [26]. In an IFN‐γ‐stimulated keratinocyte model, EGF reduced a substantial subset of inflammatory transcripts, but this was not an acne‐specific model [28].

EGF also promoted keratinocyte migration in experimental wound‐healing assays involving Kindlin‐1 and ILK/ELMO2 signaling, but these findings do not demonstrate correction of follicular hyperkeratinization or resolution of acne lesions [14, 18]. In SZ95 sebocytes, EGF stimulated proliferation and IL‐6 secretion and produced mixed effects on lipid accumulation and inflammatory gene expression, indicating that its influence on the pilosebaceous unit may not be uniformly beneficial [23].

## 4 Biological Role of EGF in Skin Physiology

### 4.1 Receptor Interaction and Downstream Pathways

EGF binds to EGFR, a transmembrane tyrosine kinase receptor expressed by epidermal keratinocytes. Ligand binding induces receptor dimerization and autophosphorylation, followed by activation of downstream pathways that include MAPK/ERK, PI3K/AKT, PLCγ/PKC, and Src‐related signaling. In keratinocytes, these pathways regulate cellular survival, proliferation, migration, differentiation, and inflammatory signaling. However, the biological response varies according to cell type, differentiation state, ligand concentration, and duration of exposure [10, 12, 15, 16, 17, 19, 20].

### 4.2 Roles in Wound Healing, Inflammation Modulation, and Keratinocyte Proliferation

Experimental studies indicate that EGF can promote keratinocyte migration and re‐epithelialization through pathways involving Kindlin‐1 and ILK/ELMO2. Additional non‐acne wound models support a role for EGF‐related interventions in tissue repair. However, these models concern cutaneous wound closure rather than follicular keratinization, comedogenesis, or resolution of active acne lesions [11, 13, 14, 18, 29, 30, 31, 32, 33].

In cultured human keratinocytes exposed to *C. acnes*, rhEGF reduced IL‐1α, IL‐8, and TNF‐α expression and attenuated TLR2/NF‐κB signaling [26]. EGF also reduced IFN‐γ‐induced inflammatory gene expression in a separate non‐acne keratinocyte model [28]. Conversely, EGFR signaling has context‐dependent effects on epidermal chemokine expression, differentiation, and barrier function, and sustained EGF exposure impaired terminal differentiation and barrier integrity in an experimental organotypic model [19, 34]. EGF should therefore not be characterized as uniformly anti‐inflammatory or barrier‐enhancing.

### 4.3 Potential Paradox Between Keratinocyte Repair and Comedogenesis

EGF is a potent mitogen that can promote keratinocyte survival, cell‐cycle progression, proliferation, and migration through EGFR‐dependent signaling. EGF exposure can also induce keratins 6 and 16, which are associated with activated and hyperproliferative keratinocyte phenotypes. These effects may be beneficial during acute wound repair, in which temporary proliferation and migration are required for re‐epithelialization [14, 15, 16, 17, 18].

However, keratinocyte proliferation cannot be assumed to be beneficial in acne‐prone follicles. Follicular hyperkeratinization involves abnormal keratinocyte proliferation, differentiation, cohesion, and desquamation, leading to microcomedone formation. Sustained EGF exposure has suppressed terminal differentiation and altered lipid‐matrix, cornified‐envelope, and tight‐junction pathways in experimental epidermal models, although EGFR activation may produce different effects under other cellular conditions. The effect of topical EGF is therefore likely to depend on exposure duration, concentration, vehicle, barrier status, and the differentiation state of the follicular epithelium [2, 3, 15, 19, 20].

At present, it is unknown whether topical EGF produces a transient repair response without worsening follicular hyperkeratinization or whether repeated exposure could promote comedogenesis in susceptible individuals. The available mechanistic studies did not reproduce the complete pilosebaceous environment, and the clinical studies did not assess microcomedone formation, follicular histology, keratinization markers, or treatment‐emergent comedonal worsening [12, 15, 18, 19, 22, 23, 35].

### 4.4 Context‐Dependent Effects on Sebocytes and Sebum Biology

Sebaceous dysfunction in acne involves more than the total quantity of sebum and includes changes in lipid composition, inflammatory activity, sebocyte differentiation, and interactions with the follicular microbiome. Therefore, a reduction in intracellular lipid accumulation cannot by itself be interpreted as a clinically beneficial sebosuppressive effect. Quantitative lipidomic studies also demonstrate that acne is associated with complex changes across several skin‐surface lipid classes rather than a uniform increase or decrease in all lipids [3, 21, 24].

In SZ95 sebocytes, EGF stimulated cellular proliferation and IL‐6 secretion and altered the expression of genes involved in steroid, retinoid, and lipid metabolism. In the presence of palmitic acid, EGF reduced intracellular lipid accumulation but increased IL‐6 secretion and enhanced hyperproliferative and inflammatory transcriptional pathways. These findings indicate that EGF may reduce selected lipid‐related responses while simultaneously promoting cellular and inflammatory changes that could be unfavorable in acne [23].

A small six‐week split‐face clinical trial reported decreased surface‐sebum output on the rhEGF‐treated side, together with improvements in inflammatory and non‐inflammatory lesion counts. However, the study did not establish whether the sebum change resulted from a direct action on sebocytes, altered follicular physiology, the formulation vehicle, or another indirect mechanism. The finding has not been independently replicated, and the available clinical studies do not provide sufficient evidence that topical EGF produces consistent or sustained sebosuppression [22, 35].

### 4.5 Potential and Uncertain Implications for Acne‐Related Care

EGF‐mediated migration and re‐epithelialization provide an indirect rationale for tissue repair after inflammatory lesions or procedures. However, evidence from acute wound healing should not be extrapolated directly to repeated application on intact acne‐prone skin. Any potential repair benefit must be balanced against the unresolved possibility of excessive proliferation, altered terminal differentiation, or impaired barrier homeostasis during sustained exposure [14, 18, 19].

Available mechanistic evidence supports only the hypothesis that topical EGF might modify acne‐associated inflammatory signaling. The *C. acnes* keratinocyte study did not evaluate bacterial killing, sebum production, comedone formation, follicular penetration, or clinical outcomes, while the IFN‐γ study used a non‐acne inflammatory stimulus. Furthermore, the sebocyte study demonstrated mixed responses, including reduced palmitic‐acid‐induced lipid accumulation but increased proliferation, IL‐6 secretion, and hyperproliferative‐inflammatory transcriptional activity [23, 26, 28].

Experimental wound models suggest that EGF‐related interventions can influence re‐epithelialization, collagen organization, and tissue remodeling [18, 29, 30, 32, 33]. These findings provide an indirect rationale for studying EGF in acne‐related repair. However, non‐acne wound closure and collagen organization cannot be interpreted as evidence that topical EGF prevents acne scars. Clinical studies have primarily evaluated established atrophic scars or post‐laser recovery rather than prospective scar prevention.

## 5 Rationale for Topical Use of EGF in Acne Treatment

### 5.1 Rationale for Topical Administration and Unresolved Exposure Questions

Topical administration permits direct application to acne‐affected skin, but acne‐specific data concerning follicular deposition, local bioavailability, systemic absorption, and optimal exposure remain limited [26]. Acne predominantly affects sebaceous‐gland‐rich areas, including the face and upper trunk [2]. Because EGF is a hydrophilic peptide of approximately 6 kDa, passive penetration through intact skin is limited and may depend on the formulation or the use of barrier‐disrupting procedures [36, 37, 38].

Short‐term active‐acne studies did not report serious treatment‐related adverse events, but together they included only 56 participants and followed treatment for six to twelve weeks. These studies were not designed to measure systemic absorption, cumulative tissue exposure, persistent epidermal proliferation, or delayed adverse outcomes. Consequently, the absence of serious reported events should be interpreted as preliminary short‐term tolerability rather than confirmation of long‐term safety [22, 35, 36].

Topical administration may theoretically reduce systemic exposure relative to systemic delivery, but no acne‐specific pharmacokinetic study has quantified circulating EGF, follicular exposure, receptor activation, or accumulation during repeated application. Reduced systemic risk therefore remains an untested assumption rather than a demonstrated advantage [22, 35, 36, 37, 38].

## 6 Formulation and Delivery Challenges of Topical EGF

Formulation is a major determinant of whether biologically active EGF can remain stable, be released from its vehicle, and reach relevant cutaneous compartments. However, the existing formulation evidence is not acne‐specific. EGF‐related delivery studies have predominantly used in vitro diffusion systems, ex vivo skin, cultured cells, or non‐acne wound models. No study has demonstrated reproducible delivery of active EGF to the pilosebaceous unit in patients with acne or linked an advanced delivery platform to improved active‐acne outcomes. The available formulation evidence is summarized in Table 2 [30, 32, 33, 37, 38].

**TABLE 2:** Critical appraisal of EGF‐specific formulation and delivery evidence.

Study and platform	Experimental model	Principal finding	Relevance and limitation
Surini et al. (2020)—rhEGF transfersomal emulgel	In vitro penetration through excised rat skin; three‐month stability testing	Increased penetration compared with non‐transfersomal emulgel; formulation‐dependent stability	EGF‐specific formulation evidence, but no human acne skin, follicular localization, biological‐effect assessment, or clinical acne outcomes [37]
Ternullo et al. (2018)—deformable hEGF liposomes	Ex vivo full‐thickness human skin and cultured fibroblasts/keratinocytes	Surface retention and sustained release; no detectable full‐thickness penetration; formulation‐dependent mitogenic activity	Human‐skin evidence, but developed for chronic wounds and did not assess acne‐prone follicles or acne outcomes [38]
Kim et al. (2018)—hyaluronate–EGF conjugate patch	Experimental chronic‐wound models	Improved stability and wound‐healing activity compared with unconjugated EGF	Non‐acne wound evidence; does not establish delivery or effectiveness in intact acne‐prone skin [32]
Montazeri et al. (2023)—EGF‐loaded chitosan nanoparticles	In vitro characterization and non‐acne animal‐wound model	Carrier‐dependent release and accelerated experimental wound repair	No acne model, follicular‐deposition assessment, or clinical evaluation [33]
Hu et al. (2025)—chemically modified mRNA encoding EGF	Diabetic‐wound model	Prolonged local EGF expression and improved experimental healing	Gene‐delivery strategy rather than conventional topical rhEGF; relevance to acne delivery and safety is uncertain [30]

Abbreviations: EGF, epidermal growth factor; hEGF, human epidermal growth factor; mRNA, messenger RNA; rhEGF, recombinant human epidermal growth factor.

### 6.1 Stability and Biological Activity

As a peptide, EGF may be affected by temperature, pH, proteolysis, aggregation, oxidation, and interactions with formulation excipients. Stability findings are therefore formulation‐specific and should not be generalized across commercial creams, gels, serums, or pharmaceutical rhEGF preparations. Available studies have not established a universal shelf life for topical EGF products used in acne [32, 37, 38].

In the transfersomal‐emulgel study, rhEGF content was assessed for three months under refrigerated and accelerated storage conditions, with greater loss observed under the higher‐temperature condition. This finding supports the importance of temperature‐controlled and product‐specific stability testing but does not establish a general post‐opening shelf life for topical EGF products [37].

Hyaluronate–EGF conjugation and other carrier‐based approaches have improved experimental stability or sustained delivery in wound‐related models. However, these systems differ from the conventional creams and ointments used in the active‐acne studies and have not been evaluated as acne treatments [32, 35, 37].

### 6.2 Skin Penetration and Localization

rhEGF is a hydrophilic peptide with a molecular mass of approximately 6 kDa, making passive passage through the intact stratum corneum difficult. Nevertheless, molecular size alone does not determine cutaneous delivery; vehicle composition, peptide stability, skin condition, application duration, and the experimental model also influence the observed result [37, 38].

Surini et al. reported that a transfersomal emulgel increased rhEGF penetration through excised rat skin compared with a non‐transfersomal emulgel, with the highest‐performing formulation producing an approximately 5.6‐fold enhancement. However, this was an in vitro rat‐skin experiment and did not assess follicular localization, biological activity within human pilosebaceous units, or acne outcomes [37].

In contrast, Ternullo et al. evaluated deformable hEGF liposomes using ex vivo full‐thickness human skin. hEGF was not detected across the complete skin thickness, and anionic liposomes primarily created a depot on the skin surface. Although the formulation increased mitogenic activity in cultured fibroblasts and keratinocytes, the study was designed for chronic‐wound therapy and did not demonstrate follicular delivery or clinical relevance to acne [38].

### 6.3 Experimental Delivery Strategies

Transfersomes and deformable liposomes are the most directly studied topical rhEGF delivery systems. They may alter peptide retention, release, and penetration compared with simple formulations, but the available studies used rat skin or ex vivo human skin and did not evaluate acne lesions or acne‐prone follicles [37, 38].

Other EGF‐related approaches include hyaluronate–EGF conjugates, chitosan nanoparticles, and chemically modified mRNA encoding EGF. These systems demonstrated improved stability, sustained biological activity, or tissue repair in experimental wound models. However, they differ substantially in composition and mechanism from conventional topical rhEGF and should not be interpreted as evidence that nanoparticle or nucleic‐acid delivery is effective or safe for active acne [30, 32, 33].

Enhanced penetration should not automatically be considered beneficial. The desired delivery profile for acne would need to provide sufficient local exposure in the relevant epidermal or follicular compartment while avoiding excessive EGFR activation, unwanted keratinocyte or sebocyte proliferation, and unnecessary systemic exposure. Delivery studies should therefore evaluate biological activity and safety alongside penetration rather than using cumulative permeation alone as evidence of therapeutic superiority [12, 15, 18, 19, 23, 36, 37, 38, 39].

## 7 Preclinical Evidence

Preclinical studies provide indirect evidence concerning EGF/EGFR signaling in keratinocytes, sebocytes, and experimental wound models. These studies suggest context‐dependent effects on inflammation, cellular migration, proliferation, and tissue repair. However, most were not conducted in acne models and should not be interpreted as direct evidence that topical EGF improves active acne lesions or prevents acne scarring in patients. Findings of in vitro studies are demonstrated in Table 3 and indirect evidence from non‐acne animal wound models in Table 4.

**TABLE 3:** Preclinical in vitro evidence potentially relevant to EGF in acne‐related research.

Model	Key finding	Relevance to acne
Human keratinocytes + *C. acnes*	rhEGF reduced IL‐1α, IL‐8, and TNF‐α expression and attenuated TLR2 expression and NF‐κB activation in C. acnes‐stimulated keratinocytes	Acne‐relevant in vitro cytokine modulation; antimicrobial activity, follicular effects, and clinical outcomes were not evaluated [26]
HaCaT keratinocytes + IFN‐γ	EGF reduced a substantial subset of IFN‐γ‐induced inflammatory transcripts in cultured keratinocytes	Demonstrates anti‐inflammatory transcriptional effects in a non‐acne inflammatory model [28]
Primary keratinocytes	EGF induced BLIMP1 expression through EGFR signaling in keratinocytes. BLIMP1 acted as a negative regulator of EGFR‐associated keratinocyte migration and inflammatory gene expression	Demonstrates regulation of keratinocyte inflammation and migration; relevance to acne remains indirect [40]
Keratinocyte migration assay	EGF activated ILK/ELMO2–RhoG→Rac1, boosting front‐rear polarity and persistent motility	Demonstrates keratinocyte migration in vitro; clinical relevance to acne‐lesion resolution is unproven [14]
SZ95 sebocytes ± palmitic acid	EGF stimulated sebocyte proliferation and IL‐6 secretion and altered steroid‐, retinoid‐, and lipid‐related gene expression. In the presence of palmitic acid, EGF reduced lipid accumulation but enhanced hyperproliferative and inflammatory transcriptional pathways	Provides acne‐relevant but context‐dependent in vitro evidence. Reduced lipid accumulation occurred alongside potentially unfavorable proliferative and inflammatory effects and should not be interpreted as simple sebosuppression [23]

Abbreviations: *C. acnes*, *Cutibacterium acnes*; EGF, epidermal growth factor; EGFR, epidermal growth factor receptor; ELMO2, engulfment and cell motility 2; HaCaT, immortalized human keratinocyte cell line; IL, interleukin; IFN‐γ, interferon gamma; ILK, integrin‐linked kinase; NF‐κB, nuclear factor kappa B; Rac1, Ras‐related C3 botulinum toxin substrate 1; rhEGF, recombinant human epidermal growth factor; RhoG, Ras homolog gene family member G; TLR2, toll‐like receptor 2; TNF‐α, tumor necrosis factor alpha.

**TABLE 4:** Indirect evidence from non‐acne animal wound models relevant to EGF‐mediated tissue repair.

Animal model	Intervention	Outcome	Implication for acne sequelae
Full‐thickness dorsal wounds, mice	Topical HB‐EGF in heparin coacervate	2× faster closure, thicker granulation tissue, richer neovessels	Provides indirect evidence concerning tissue repair in a non‐acne wound model [31]
Mouse wounds	Skin‐permeable LMWP‐EGF nanocomplex	25% faster re‐epithelialization vs. native EGF	Demonstrates enhanced delivery and healing in a non‐acne wound model [29]
Type‐1‐diabetic mice	Chemically‐modified mRNA encoding EGF	Day‐6 wounds already > 80% closed; collagen better organized	Provides proof‐of‐concept for prolonged EGF expression in diabetic wounds [30]

Abbreviations: EGF, epidermal growth factor; HB‐EGF, heparin‐binding epidermal growth factor‐like growth factor; low‐molecular‐weight protamine‐conjugated epidermal growth factor; mRNA, messenger RNA.

The heparin‐binding EGF (HB‐EGF) study was retained as indirect evidence concerning controlled delivery of an EGFR‐family ligand in cutaneous wound repair. However, HB‐EGF should not be assumed to be pharmacologically or clinically equivalent to topical EGF in acne [31].

## 8 Clinical Evidence of Topical EGF in Acne

The strength of the clinical evidence was interpreted according to study design, sample size, use of controls and blinding, outcome assessment, treatment duration, and applicability to either active acne or established acne scars. The clinical literature was divided according to indication because treatment of active acne lesions, management of established acne scars, and post‐procedural recovery represent clinically distinct applications. Direct evidence for topical EGF in active acne is limited to small, short‐term split‐face trials. Additional studies have evaluated topical EGF‐containing formulations for established atrophic acne scars or EGF as an adjunct to fractional laser procedures. Evidence from these latter settings should not be extrapolated directly to the treatment of active AV. A comparative summary and critical appraisal of the design, sample size, interventions, principal findings, and limitations of the available human studies are presented in Table 5.

**TABLE 5:** Critical appraisal of human clinical evidence concerning topical EGF in acne‐related care.

Indication and study	Design/sample	Intervention and comparator	Main findings	Principal limitations
Active acne—Kim et al. (2014)	Randomized, double‐blind, split‐face; *n* = 20; 6 weeks	rhEGF cream versus vehicle	Reported reductions in inflammatory and non‐inflammatory lesion counts and surface‐sebum output	Very small sample; six‐week treatment; no active comparator; no pharmacokinetic assessment or long‐term proliferative safety follow‐up; limited generalizability [22]
Active acne and scars—Kim et al. (2022)	Prospective, single‐blinded, split‐face; *n* = 36; 12 weeks	EGF ointment versus vehicle	Reported improvements in lesion counts, IGA, scar‐related, and exploratory histological outcomes	Small, single‐center study; no active comparator; 12‐week follow‐up; exploratory histology; not designed to assess cumulative proliferative or oncologic safety [35]
Atrophic scars—Seidel and Moy (2015)	Uncontrolled pilot; 9 enrolled, 8 completed; 12 weeks	Full‐face EGF serum; no control	Modest investigator and patient‐reported improvement	No comparator; extremely small sample; subjective outcomes [41]
Atrophic scars in skin of color—Stoddard et al. (2017)	Uncontrolled pilot; 12 enrolled, 11 completed; 12 weeks	Full‐face EGF serum; no control	Improvement in IGA, Goodman grade, photographs, and self‐assessment	No control; small sample; expectation and photographic bias possible [42]
Post‐laser scar treatment—Ratanapokasatit and Sirithanabadeekul (2022)	Randomized split‐face; 23 enrolled, 21 completed	Laser plus EGF versus laser plus placebo	Small scar‐assessment advantage at 3 months; no wound‐healing difference	Small sample; short follow‐up; procedural indication only [43]
Post‐laser scar treatment—Peng et al. (2024)	Randomized split‐face; *n* = 15	Laser plus rhEGF versus laser plus placebo	No significant additional benefit in principal scar outcomes; pore/pigment benefits	Very small sample; both sides received effective laser; limited additive effect [44]
Post‐laser scar treatment—Wang et al. (2024)	Retrospective comparison; *n* = 105	Laser plus rhEGF versus laser alone	Better reported recovery, barrier, inflammatory, and satisfaction outcomes	Nonrandomized; retrospective; confounding and selection bias [45]
Procedural scar evidence—Zhou et al. (2026)	Systematic review/meta‐analysis; 12 RCTs; *n* = 1044	Laser plus rhEGF versus laser alone	Pooled improvement in scar scores and some recovery/safety outcomes	All trials from China; heterogeneity; very‐low‐to‐moderate certainty; no long‐term safety or malignancy outcomes; not active acne [46]

Abbreviations: EGF, epidermal growth factor; IGA, Investigator's Global Assessment; RCT, randomized controlled trial; rhEGF, recombinant human EGF.

### 8.1 Topical EGF for Active Acne Vulgaris

Kim et al. evaluated 36 patients with mild‐to‐moderate AV in a 12‐week prospective, split‐face, single‐blinded study. Participants applied EGF ointment to one facial side and vehicle ointment to the contralateral side twice daily. Acne‐lesion counts reportedly improved from week 4, whereas Investigator's Global Assessment scores improved from week 8. Biopsy findings also suggested changes in inflammatory, proliferative, and extracellular‐matrix‐related markers. However, the study was small, single‐center, short‐term, and did not include an established active‐acne treatment as a comparator. Its split‐face design and limited sample also restrict generalizability and do not establish effectiveness in broader clinical populations [35].

The earlier study enrolled 20 adults with mild‐to‐moderate facial acne in a six‐week randomized, double‐blind, vehicle‐controlled split‐face trial. Participants applied rhEGF cream to one facial side and vehicle cream to the other twice daily. At six weeks, inflammatory lesions were reduced by 33.5% and non‐inflammatory lesions by 25.4% on the rhEGF‐treated side; surface‐sebum output also decreased. Nevertheless, the small sample, short treatment period, single‐country setting, vehicle‐only comparator, and absence of longer post‐treatment follow‐up substantially limit the certainty and external validity of the findings [22].

The 2014 trial also measured surface‐sebum output and reported a reduction on the rhEGF‐treated side over six weeks. Although this finding suggests a possible effect on sebaceous activity, the small sample, short treatment duration, and absence of detailed lipid‐composition or sebaceous‐gland assessments limit mechanistic interpretation. Moreover, the result should be considered alongside the mixed in vitro sebocyte findings, in which EGF reduced palmitic‐acid‐induced lipid accumulation but increased proliferation, IL‐6 secretion, and inflammatory transcriptional activity [22, 23].

Taken together, direct clinical evidence for active acne comprises only 56 participants across two short‐term split‐face studies. Both studies used vehicle controls rather than guideline‐supported active comparators, and neither was a large parallel‐group trial. Although both studies reported within‐study improvements in acne‐related outcomes, these findings should be regarded as preliminary efficacy signals rather than evidence of clinically established benefit. The small samples, short follow‐up periods, restricted populations, heterogeneous formulations, vehicle‐only comparators, and lack of independent large‐scale confirmation prevent conclusions regarding clinically meaningful efficacy, comparative effectiveness, optimal dosing, durability, or routine use [9, 22, 35].

Neither active‐acne trial was designed to assess microcomedone development, chronic follicular hyperkeratinization, or long‐term comedogenic risk. Molecular and histological observations in the 2022 study provide exploratory mechanistic information but cannot establish the long‐term effects of repeated EGF exposure on acne‐prone follicles [12, 15, 18, 19, 22, 35].

### 8.2 Topical EGF for Established Atrophic Acne Scars

Two uncontrolled pilot studies evaluated topical synthetic EGF‐containing serum for established atrophic acne scars. In one single‐center study, nine patients with grade II–IV atrophic acne scars applied the serum twice daily for 12 weeks, and eight completed the study. Modest improvements were reported using clinical grading, standardized photography, investigator assessment, and patient self‐assessment. However, the absence of a control group and the very small sample size preclude firm conclusions regarding efficacy [41].

A second 12‐week pilot study enrolled 12 participants with Fitzpatrick skin types IV–V and atrophic acne scars, of whom 11 completed treatment. Improvements in investigator and patient assessments were reported, but this study was also small, uncontrolled, and reliant predominantly on clinical grading and photography [42]. These studies relate to established acne scars and should not be interpreted as evidence that topical EGF treats active acne or prevents scar formation.

Collectively, these two studies provide only low‐certainty preliminary evidence for topical EGF serum in established atrophic acne scars. Both were single‐center, uncontrolled before‐and‐after studies with fewer than 12 completers, and both relied substantially on investigator grading, photography, and patient self‐assessment. Without a vehicle or active control group, spontaneous variation, photographic conditions, expectation effects, and concurrent skincare cannot be excluded as explanations for part of the observed improvement [41, 42].

### 8.3 EGF as an Adjunct to Acne‐Scar Procedures

Several studies have evaluated topical recombinant human EGF after ablative fractional carbon dioxide laser treatment for atrophic acne scars. A split‐face trial reported greater improvement in scar appearance and pigmentation on the EGF‐treated side than on the placebo‐treated side, without reported allergic reactions [43]. Other studies have reported improvements in scar‐related clinical outcomes, inflammatory measures, or post‐procedural barrier recovery when fractional carbon dioxide laser treatment was combined with recombinant human EGF gel [44, 45].

These findings concern EGF as a post‐procedural adjunct for established acne scars. They do not demonstrate that topical EGF alone treats active acne lesions, prevents the development of scars, or provides benefit comparable with established acne medications. Interpretation is further limited by heterogeneous laser protocols, EGF formulations, outcome measures, treatment durations, and follow‐up periods.

### 8.4 Formulations, Dosing, and Treatment Duration According to Indication

In the principal active‐acne study, EGF ointment was applied twice daily for 12 weeks, with assessments conducted at four‐week intervals. Acne‐lesion improvement was reported from week 4, whereas changes in scar‐related outcomes required a longer treatment period. Acne‐specific EGF concentration and dose reporting remain inconsistent across the available studies [35]. The uncontrolled acne‐scar pilot studies used topical synthetic EGF‐containing serum twice daily for 12 weeks [41, 42].

A randomized split‐face study enrolled 23 participants with atrophic acne scars, of whom 21 completed three monthly fractional carbon dioxide laser sessions. Topical EGF or placebo was applied twice daily for seven days after each procedure. At three months, modest between‐side differences favored EGF in blinded physician grading and Antera 3D analysis, although wound‐healing measures did not differ between groups. The small sample and limited follow‐up restrict the certainty of these findings [43].

Peng et al. subsequently conducted a randomized split‐face study involving 15 patients who underwent three fractional carbon dioxide laser treatments. Both facial sides improved substantially, but rhEGF did not significantly enhance the principal scar‐related outcomes compared with placebo. Between‐side advantages were limited mainly to pore number, epidermal pigmentation, and aspects of post‐procedural recovery. This study therefore does not demonstrate a clear additive effect of rhEGF on overall scar correction [44].

Wang et al. retrospectively evaluated 105 patients, of whom 51 received fractional carbon dioxide laser alone and 54 received laser combined with rhEGF gel. The combination group showed more favorable barrier, inflammatory, recovery‐time, satisfaction, and adverse‐event outcomes. However, the retrospective, nonrandomized design creates substantial potential for selection bias, baseline imbalance, treatment‐allocation bias, and uncontrolled confounding. The findings should therefore be interpreted as supportive observational evidence rather than confirmation from a randomized trial [45].

A 2026 systematic review and meta‐analysis included 12 randomized trials involving 1044 patients and compared fractional carbon dioxide laser plus rhEGF with laser treatment alone for facial atrophic acne scars. The pooled analysis favored combination treatment for ECCA and Vancouver Scar Scale scores and suggested shorter crusting duration and a lower risk of hyperpigmentation. However, all included studies were conducted in China, treatment protocols and follow‐up periods were heterogeneous, and GRADE certainty ranged from very low to moderate. These findings provide additional, although still limited, support for further investigation of rhEGF in the procedural management of established acne scars; they do not support topical EGF as monotherapy or as treatment for active AV [46].

Because the available studies evaluated different clinical indications and formulations, their dosing regimens should not be considered interchangeable. No standardized concentration, vehicle, application frequency, or duration has been established for either active acne or acne‐scar management.

### 8.5 Safety and Tolerability in the Available Clinical Studies

No serious treatment‐related adverse events were reported in the small, short‐term studies of active acne, topical acne‐scar serum, or post‐laser rhEGF treatment. Nevertheless, adverse‐event monitoring was limited, sample sizes were generally small, and treatment and follow‐up periods were brief. In procedural studies, erythema, irritation, crusting, and barrier changes may also be difficult to attribute specifically to rhEGF because fractional laser treatment itself produces transient cutaneous effects [22, 35, 41, 42, 43, 44, 45, 46].

These findings support only preliminary short‐term tolerability. The available studies were not designed to evaluate pharmacokinetics, cumulative EGFR stimulation, persistent epidermal hyperplasia, treatment‐emergent proliferative lesions, or rare and delayed adverse outcomes. No active‐acne study included sufficiently long follow‐up to characterize the safety of repeated or maintenance use [22, 35, 36].

### 8.6 Long‐Term Proliferative and Oncologic Uncertainty

EGF promotes cellular survival, proliferation, and migration through EGFR‐dependent signaling. These actions are biologically appropriate during short‐term tissue repair but raise a theoretical concern that prolonged or excessive exposure could stimulate undesirable proliferation, particularly in tissue containing pre‐existing atypical cells. Experimental studies demonstrating hyperproliferation‐associated keratins and altered terminal differentiation reinforce the need for dose‐ and duration‐specific safety assessment [10, 12, 15, 19].

A theoretical proliferative concern should not be interpreted as evidence that topical EGF causes cancer. Reviews of therapeutic EGF use have not established a causal association with malignancy; however, the available populations, treatment indications, sample sizes, and follow‐up periods are inadequate to exclude rare or delayed risks. Evidence obtained from short‐term wound‐healing or procedural use also cannot establish the safety of repeated application to intact acne‐prone skin over months or years [11, 36, 39].

### 8.7 Overall Certainty of the Clinical Evidence

Direct evidence for active acne remains very limited. The two available studies included only 56 participants in total, used short‐term split‐face designs, and did not compare EGF with an established active acne therapy. Consequently, they provide preliminary efficacy signals rather than sufficient evidence for routine clinical use [9, 22, 35].

Evidence for topical EGF serum used alone on established atrophic scars is weaker because it is based on two very small uncontrolled pilot studies. Evidence for rhEGF after fractional carbon dioxide laser is broader and now includes randomized trials and a 2026 meta‐analysis; however, heterogeneity, geographic concentration, variable treatment protocols, and predominantly very‐low‐to‐moderate certainty limit generalizability. Procedural scar evidence should not be extrapolated to active acne or topical EGF monotherapy [41, 42, 43, 44, 45, 46].

Across all indications, the literature lacks adequately powered multicenter parallel‐group trials, standardized EGF concentrations and vehicles, consistent outcome definitions, active comparators, prespecified primary outcomes, and long‐term follow‐up. The available evidence therefore remains insufficient to establish comparative effectiveness, optimal treatment parameters, durability, or long‐term safety.

No included study directly compared topical EGF with a guideline‐supported active‐acne treatment; therefore, claims of superiority, non‐inferiority, improved tolerability, or treatment replacement are not supported by the current evidence [9, 22, 35].

## 9 Contextual Comparison With Guideline‐Supported Acne Therapies

Available evidence is insufficient to establish that topical EGF is superior, non‐inferior, or equivalent to guideline‐supported acne therapies. Topical EGF has not been compared directly with retinoids, benzoyl peroxide, topical antibiotics, or fixed‐combination treatments. The following discussion is therefore contextual and mechanistic rather than a comparison of demonstrated clinical effectiveness [9].

Topical retinoids are guideline‐supported treatments for active acne because of their comedolytic and anti‐inflammatory effects. Local erythema, dryness, scaling, and irritation may affect adherence, but their efficacy and clinical role are supported by substantially broader evidence than is currently available for topical EGF. Because no direct comparison has been conducted, the relative efficacy, tolerability, and long‐term benefit of topical EGF versus topical retinoids remain unknown [6, 9].

Benzoyl peroxide is a guideline‐supported treatment with antimicrobial activity against *C. acnes* and no established mechanism for inducing bacterial resistance. Irritation, dryness, and erythema may occur, particularly during treatment initiation. Topical EGF has not been compared directly with benzoyl peroxide, and no conclusion can currently be made regarding relative lesion reduction, speed of response, tolerability, adherence, or relapse prevention [9].

Topical antibiotics are used primarily as components of combination regimens because monotherapy may contribute to antimicrobial resistance. EGF is not an antimicrobial agent, and its reported effects on keratinocyte cytokine signaling would not be expected to exert conventional antibiotic selection pressure. Nevertheless, the absence of an antibiotic‐resistance mechanism does not establish equivalent clinical efficacy, and topical EGF has not been shown to replace antibiotic‐containing regimens or established non‐antibiotic therapies [8, 9, 26].

### 9.1 Potential for Combination Therapy

Combination of topical EGF with retinoids, benzoyl peroxide, topical antibiotics, or fixed‐combination acne treatments has not been evaluated adequately in controlled clinical trials. Consequently, potential complementary effects, sequencing, irritation profiles, dose requirements, and effects on adherence remain unknown. These combinations should be considered research hypotheses rather than recommended treatment strategies.

The principal clinical evidence for EGF‐containing combination treatment concerns rhEGF applied after fractional carbon dioxide laser treatment for established atrophic acne scars. Individual studies have produced mixed findings: some reported modest benefits in scar assessment or post‐procedural recovery, whereas one small randomized split‐face study found no significant additional improvement in the principal scar outcomes [43, 44, 45].

A 2026 systematic review and meta‐analysis of 12 randomized trials involving 1044 patients reported pooled benefits for some scar, recovery, and safety outcomes when fractional carbon dioxide laser was combined with rhEGF. However, certainty ranged from very low to moderate; all included studies were conducted in China, and these findings apply to procedural management of established atrophic scars rather than active acne treatment [46].

### 9.2 Evidence‐Based Clinical Positioning by Indication

#### 9.2.1 Active Acne Vulgaris

No evidence‐based clinical role has been established for topical EGF as monotherapy, replacement therapy, maintenance therapy, or routine adjunctive treatment for active acne. Direct evidence is limited to two small, short‐term, vehicle‐controlled split‐face studies, neither of which compared EGF with a guideline‐supported active treatment. Topical EGF should therefore remain investigational in this setting and should not replace retinoids, benzoyl peroxide, or appropriate combination regimens [9, 22, 35].

#### 9.2.2 Prevention of Acne Scarring

No prospective study has evaluated whether topical EGF reduces the incidence or severity of new scars in patients receiving treatment for active acne. Histological observations, non‐acne wound‐healing studies, treatment of established scars, and post‐laser recovery studies cannot establish a scar‐preventive effect. Topical EGF therefore has no currently supported role in acne‐scar prevention [18, 30, 31, 32, 33, 35, 41, 42, 43, 44, 45, 46].

#### 9.2.3 Topical Treatment of Established Atrophic Scars

Evidence for EGF‐containing serum used without a procedural intervention is limited to two uncontrolled pilot studies with fewer than 12 completers each. These studies provide insufficient evidence to define topical EGF serum as an established treatment for atrophic acne scars [41, 42].

#### 9.2.4 Post‐Procedural Treatment of Established Atrophic Scars

The comparatively broader clinical evidence concerns rhEGF used after fractional carbon dioxide laser treatment. Randomized split‐face studies, a retrospective comparison, and a recent meta‐analysis suggest possible benefits for selected scar, pigmentation, barrier‐recovery, or post‐procedural outcomes. Nevertheless, findings are inconsistent, treatment protocols are heterogeneous, and certainty ranges from very low to moderate. This indication represents the most plausible research niche for rhEGF but not an established standard of care [43, 44, 45, 46].

Overall, the current evidence does not support a defined position for topical EGF within the routine active‐acne treatment algorithm. Its most defensible potential role is as an investigational post‐procedural adjunct for established atrophic acne scars, provided that future trials demonstrate clinically meaningful incremental benefit over the procedure alone [9, 43, 44, 45, 46].

## 10 Limitations and Knowledge Gaps

### 10.1 Limitations of the Mechanistic Evidence

Most proposed mechanisms are derived from cultured keratinocytes, immortalized sebocytes, non‐acne wound models, EGFR‐inhibition models, or experiments involving EGFR ligands other than EGF. These systems do not reproduce the complete pilosebaceous microenvironment, microbial community, immune response, follicular keratinization, or topical pharmacokinetics of active acne. Accordingly, the direction and magnitude of EGF effects observed in these models may differ from those occurring after topical application to acne‐prone human skin [14, 18, 19, 23, 26, 28, 31, 34].

A further uncertainty concerns the proliferative effects of EGF on follicular keratinocytes. Although transient proliferation and migration may facilitate tissue repair, sustained EGFR activation can induce hyperproliferation‐associated keratins and suppress aspects of terminal epidermal differentiation in experimental systems. Because the available studies did not evaluate microcomedone formation or chronic exposure in acne‐prone follicles, the possibility that topical EGF could aggravate follicular hyperkeratinization cannot currently be excluded [12, 15, 19, 20, 22, 35].

Evidence concerning the effects of EGF on sebaceous‐gland biology is particularly limited. The principal mechanistic study used immortalized SZ95 sebocytes under controlled in vitro conditions, including exposure to palmitic acid, and did not reproduce the complete human pilosebaceous environment. Although one short‐term clinical trial reported reduced surface‐sebum output, it did not evaluate sebaceous‐gland histology, sebum lipid composition, oxidized lipids, long‐term sebocyte activity, or the relationship between sebum changes and clinical response. Consequently, the net effect of topical EGF on sebaceous‐gland function remains uncertain [22, 23].

### 10.2 Limited Number of High‐Quality Clinical Studies

Few controlled trials exist for topical EGF in acne treatment. The clinical evidence is generally derived from small studies, ranging from very small uncontrolled scar pilots to split‐face active‐acne studies involving 20 and 36 participants. Clinical study quality varies substantially by indication. The two active‐acne studies were small, short‐term split‐face vehicle‐controlled trials, whereas the two topical acne‐scar serum studies were uncontrolled pilots with fewer than 12 completers. Procedural scar studies include randomized split‐face trials and a retrospective comparative study, but they remain heterogeneous in intervention, outcome measurement, and follow‐up. A recent meta‐analysis provides broader procedural evidence, although the certainty of most pooled outcomes was very low to moderate and applicability outside the studied populations remains uncertain [22, 35, 41, 42, 43, 44, 45, 46].

The absence of active‐comparator trials is a major limitation because vehicle‐controlled improvement does not establish whether topical EGF provides clinically meaningful benefit relative to standard acne therapies [9, 22, 35].

### 10.3 Undefined Target Population and Clinical Niche

No study has prospectively identified a patient subgroup for whom topical EGF offers a particular advantage. The available trials do not establish that patients who cannot tolerate retinoids, patients with barrier dysfunction, individuals with inflammatory rather than comedonal acne, or patients considered at high risk of scarring derive greater benefit from EGF. Proposing use in any of these groups would therefore be speculative [9, 22, 35].

The clinical indication also determines the appropriate comparator and outcome. Active‐acne studies require comparison with established acne therapies and acne‐specific lesion outcomes, scar‐prevention studies require prospective assessment of new scar formation, established‐scar studies require validated scar outcomes, and post‐procedural studies must demonstrate benefit beyond the procedure and vehicle alone. Combining these indications obscures the clinical question and prevents identification of a meaningful treatment niche [9, 22, 35, 41, 42, 43, 44, 45, 46].

### 10.4 Limitations of the Review Process

This review has several methodological limitations. First, the search was restricted to PubMed‐indexed English‐language publications. Relevant studies indexed only in other databases, non‐English‐language studies, conference abstracts, regulatory documents, dissertations, and other gray literature may therefore have been missed. Restricting inclusion to PubMed improved consistency and allowed exclusion of non‐indexed and retracted material, but it does not ensure complete retrieval of all potentially relevant evidence.

Second, this was a narrative rather than a systematic review. No prospective protocol was registered, and no formal study‐level risk‐of‐bias instrument was applied across the heterogeneous evidence base. Although clinical studies were critically appraised according to their design and principal limitations, descriptive appraisal cannot provide the same standardized assessment as study‐design‐specific risk‐of‐bias tools.

Third, the evidence encompassed active acne, established atrophic scars, post‐procedural treatment, keratinocyte and sebocyte experiments, non‐acne wound models, and formulation studies. This heterogeneity prevented quantitative pooling and increased the possibility of interpretive selection when determining which indirect studies were relevant to acne‐related care.

Finally, publication bias cannot be excluded. Small studies reporting favorable clinical or wound‐healing outcomes may be more likely to be published than studies reporting neutral or unfavorable findings. The conclusions should therefore be interpreted as a critical narrative synthesis of the available literature rather than a definitive estimate of efficacy, comparative effectiveness, or safety.

### 10.5 Lack of Standardized Formulations and Dosing Regimens

No standardized EGF concentration, vehicle, application frequency, or duration has been established for active acne. Moreover, the formulations used in clinical studies cannot be assumed to be equivalent because differences in peptide identity, biological potency, excipients, stability, release, and cutaneous deposition may affect both efficacy and safety. The available advanced‐delivery studies are preclinical and have not demonstrated follicular delivery or clinical effectiveness in acne [22, 30, 32, 33, 35, 37, 38].

Measurements of increased permeation in rat skin, surface retention in ex vivo human skin, or accelerated repair in wound models do not establish clinically meaningful bioavailability in acne‐prone pilosebaceous units. Consequently, the relationship between formulation characteristics, local EGF exposure, receptor activation, and clinical response remains unknown [30, 32, 33, 37, 38].

### 10.6 Uncertainty Regarding Long‐Term Safety and Efficacy

Active‐acne studies evaluated topical EGF for only six to twelve weeks, and the available acne‐scar studies also used relatively short treatment and follow‐up periods. None was designed to assess systemic pharmacokinetics, cumulative EGFR activation, persistent epidermal hyperplasia, delayed comedogenic effects, treatment‐emergent neoplasia, or outcomes after prolonged maintenance use. The absence of reported serious adverse events in these small studies therefore cannot exclude uncommon or delayed risks [22, 35, 36, 39, 41, 42, 43, 44, 45, 46].

EGF's mitogenic activity creates a biologically plausible safety concern, but current evidence does not demonstrate that therapeutic topical EGF causes cutaneous or systemic malignancy. The principal limitation is insufficient evidence rather than evidence of confirmed carcinogenicity. Both exaggerated reassurance and unsupported claims of oncogenic harm should therefore be avoided [10, 11, 12, 15, 36, 39].

### 10.7 Regulatory Classification and Product‐Quality Considerations

The regulatory classification of an EGF‐containing product is jurisdiction‐ and product‐specific and may depend on the identity and source of the active ingredient, its concentration, formulation, route of administration, intended indication, labeling, promotional claims, and conditions of use. A preparation marketed as a cosmetic in one jurisdiction may be classified differently in another, and the presence of EGF, rhEGF, or an EGF‐related peptide alone does not determine whether the product is regulated as a cosmetic, medicinal product, biologic, or another product category. Accordingly, topical EGF should not be described universally as an unauthorized drug or as categorically outside the scope of cosmetics [36, 39].

Cosmetic preparations containing sh‐oligopeptide‐1 should not automatically be considered biologically, clinically, or regulatorily equivalent to pharmaceutical‐grade rhEGF formulations. Products may differ in peptide sequence, purity, concentration, biological potency, excipients, stability, manufacturing controls, and intended indication, all of which may influence exposure, efficacy, and safety. No standardized medicinal EGF formulation has been established for acne treatment, and the authorization or approval status of a specific product for acne should be verified within the relevant jurisdiction rather than inferred from the status of EGF‐containing products elsewhere [36, 37, 39, 47].

Future clinical studies should report the regulatory category, manufacturer, peptide identity, concentration, biological‐activity assay, formulation characteristics, intended indication, and approval status of the tested product. Regulatory and safety conclusions should remain specific to the formulation and use studied and should not be generalized to all products marketed as containing EGF or EGF‐related peptides [36, 37, 39, 47].

## 11 Future Directions

Large, adequately powered randomized controlled trials are required before topical EGF can be considered for routine acne‐related care. Studies of active acne should use standardized EGF formulations, clearly reported concentrations, parallel‐group designs, appropriate vehicle and guideline‐supported comparators, validated inflammatory and non‐inflammatory lesion counts, Investigator's Global Assessment outcomes, patient‐reported outcomes, and longer safety follow‐up.

Studies concerning active acne should be designed and interpreted separately from studies of established acne scars or post‐procedural recovery. Formulation research should determine whether EGF can achieve reproducible delivery to relevant epidermal or follicular compartments without promoting undesirable hyperproliferation or impaired terminal differentiation.

Future studies should specifically evaluate whether topical EGF influences comedogenesis. In addition to conventional inflammatory and non‐inflammatory lesion counts, trials should assess new comedone formation, follicular keratinization, keratinocyte proliferation and differentiation markers, treatment‐emergent acne worsening, and changes after treatment discontinuation. Dose‐ranging studies are also required to determine whether short or intermittent exposure can support repair without producing undesirable sustained EGFR activation.

Future clinical trials should prospectively evaluate sebaceous‐gland outcomes, including surface‐sebum excretion rate, lipid composition, lipid oxidation, sebocyte‐related biomarkers, and their relationship to inflammatory and non‐inflammatory lesion changes. Studies should also determine whether any reduction in sebum is sustained, dose‐dependent, vehicle‐dependent, and accompanied by improvement rather than increased inflammatory or hyperproliferative signaling.

Future active‐acne trials should include both vehicle and guideline‐supported active comparators, such as a topical retinoid or an appropriate fixed‐combination regimen. Non‐inferiority or superiority hypotheses, clinically meaningful margins, adverse‐event definitions, adherence outcomes, and rescue‐treatment criteria should be prespecified.

Future studies should prespecify the clinical role being tested rather than broadly enrolling participants under the combined category of “acne and acne scars.” Separate trials are required for active‐acne monotherapy, adjunctive active‐acne treatment, prevention of new scars, topical treatment of established scars, and post‐procedural recovery.

Studies intended to establish an active‐acne niche should identify the proposed target population and demonstrate incremental benefit over guideline‐supported treatment. Hypothesized subgroups, including patients with poor tolerability of conventional topical therapies or impaired barrier function, should be evaluated prospectively rather than assumed to benefit.

Post‐procedural trials should determine whether rhEGF provides a clinically meaningful advantage beyond fractional laser treatment alone. Prespecified outcomes should include validated scar scores, recovery time, pigmentary complications, patient‐reported benefit, cost, and whether any improvement persists after treatment discontinuation.

Long‐term safety studies should include treatment periods and post‐treatment surveillance substantially longer than the six‐to‐twelve‐week periods used in existing active‐acne trials. Safety outcomes should include persistent erythema or irritation, acne worsening, new comedone formation, epidermal or follicular hyperplasia, new or changing cutaneous lesions, and adverse events occurring after treatment discontinuation.

Pharmacokinetic and pharmacodynamic studies should measure local tissue exposure, systemic absorption, EGFR activation, dose–response relationships, and the duration of biological activity after application. Where ethically and clinically appropriate, biopsy‐based studies could evaluate proliferation and differentiation markers during repeated use.

Future trials should prespecify exclusion or stratification criteria for individuals with current or recent cutaneous malignancy, premalignant lesions, or conditions associated with abnormal epidermal proliferation. Longer‐term registries or structured post‐marketing surveillance would be required to identify uncommon or delayed events that cannot be detected in small randomized trials.

Future formulation studies should use standardized pharmaceutical‐grade rhEGF and report peptide sequence, concentration, purity, biological potency, excipients, particle size, encapsulation efficiency, release kinetics, storage conditions, and stability throughout the study period.

Delivery should be evaluated in human acne‐prone skin using methods capable of distinguishing surface retention, interfollicular epidermal deposition, follicular localization, dermal penetration, and systemic absorption. Demonstration of increased cumulative permeation alone should not be considered sufficient evidence of successful acne‐targeted delivery.

Clinical trials should compare clearly characterized formulations and determine whether delivery differences correspond to acne‐lesion outcomes, local EGFR activation, irritation, comedogenesis, sebocyte responses, and proliferative safety.

## 12 Conclusions

Topical EGF is a biologically plausible but clinically unestablished investigational approach in acne‐related care. Current evidence is insufficient to demonstrate clinically meaningful efficacy or to support its use as monotherapy, replacement therapy, maintenance therapy, or routine adjunctive treatment for active AV. It has also not been shown to prevent acne‐scar formation. Evidence for topical EGF serum used alone on established atrophic scars is limited to very small uncontrolled studies. The comparatively broader evidence concerns rhEGF used after fractional laser treatment for established atrophic scars, but this application also remains investigational.

These clinical applications should be considered separately. Evidence from wound‐healing models, acne‐scar procedures, and post‐procedural recovery cannot establish efficacy against active inflammatory or comedonal acne. Potential anti‐inflammatory and tissue‐repair effects must also be balanced against the unresolved possibility that prolonged EGFR stimulation could promote keratinocyte hyperproliferation or disrupt terminal differentiation in acne‐prone skin. The effect of topical EGF on sebaceous‐gland activity also remains unresolved because a preliminary clinical reduction in surface‐sebum output contrasts with mixed proliferative, inflammatory, and lipid‐related responses observed in cultured sebocytes. Topical EGF has not been compared directly with guideline‐supported acne therapies and should not currently be considered an alternative or clinically established adjunct for active acne. Although no causal association between therapeutic topical EGF and malignancy has been demonstrated, the existing short‐term studies are insufficient to establish the safety of repeated or prolonged application, and its mitogenic activity warrants dedicated long‐term surveillance. Advanced EGF delivery systems remain preclinical and have not established targeted follicular delivery, acne‐specific bioavailability, or clinical benefit in active acne. Accordingly, post‐procedural treatment of established atrophic acne scars represents the most plausible area for further clinical investigation, but no routine clinical niche can currently be recommended. These conclusions should also be interpreted in light of the narrative‐review design, the PubMed‐only English‐language search, and the absence of formal study‐level risk‐of‐bias assessment or quantitative synthesis. Until adequately powered trials demonstrate clinically meaningful incremental benefit, reproducible safety, and effectiveness relative to appropriate active comparators, topical EGF should not be considered a standard therapy for active acne vulgaris or acne‐related sequelae.

## Author Contributions

Conceptualization and study validation: N.G., G.Z., G.R.R. Implementation and supervision: N.G., G.Z., G.R.R., H.F., R.P., A.E., M.S., O.M. Writing and reviewing: N.G., G.Z., G.R.R., H.F., R.P., A.E., M.S., O.M. All authors have read and approved the final version of the manuscript.

## Funding

The authors have nothing to report.

## Ethics Statement

The authors have nothing to report.

## Consent

The authors have nothing to report.

## Conflicts of Interest

The authors declare no conflicts of interest.

## Supporting information


**Table S1:** Complete PubMed search strategies.

## Data Availability

Data sharing is not applicable to this article as no datasets were generated or analyzed during the current study.
