# Supplementary material for: Topical Epidermal Growth Factor in Acne Vulgaris and Acne‐Related Sequelae: Mechanistic Rationale, Clinical Evidence, and Formulation Challenges
Source: J Cosmet Dermatol. 2026 Sep 11;25(9):e71189. doi: 10.1111/jocd.71189 (PMC13569868; doi:10.1111/jocd.71189)
Supplement: Supplementary file 1 [file JOCD-25-e71189-s001.docx]

Supplementary Table S1: Complete PubMed search strategies

| Search | Purpose | Complete PubMed search string |
| --- | --- | --- |
| S1 | Core clinical evidence: active acne, acne scars, and topical EGF | (("Acne Vulgaris"[Mesh] OR "acne vulgaris"[tiab] OR acne[tiab] OR "acne scar"[tiab] OR "acne scars"[tiab] OR "atrophic acne scar"[tiab] OR "atrophic acne scars"[tiab]) AND ("Epidermal Growth Factor"[Mesh] OR "epidermal growth factor"[tiab] OR EGF[tiab] OR "recombinant human epidermal growth factor"[tiab] OR rhEGF[tiab] OR "sh-oligopeptide-1"[tiab])) AND english[lang] AND ("1900/01/01"[dp] : "2026/06/30"[dp]) |
| S2 | EGF/EGFR signaling in keratinocytes and inflammatory models | (("Epidermal Growth Factor"[Mesh] OR "Receptor, Epidermal Growth Factor"[Mesh] OR "epidermal growth factor"[tiab] OR EGF[tiab] OR EGFR[tiab] OR rhEGF[tiab]) AND ("Keratinocytes"[Mesh] OR keratinocyte*[tiab]) AND ("Acne Vulgaris"[Mesh] OR acne[tiab] OR "Cutibacterium acnes"[tiab] OR "Propionibacterium acnes"[tiab] OR inflammation[tiab] OR inflammatory[tiab] OR cytokine*[tiab] OR TLR2[tiab] OR "NF-kappa B"[tiab] OR "interferon gamma"[tiab] OR IFN-gamma[tiab])) AND english[lang] AND ("1900/01/01"[dp] : "2026/06/30"[dp]) |
| S3 | Sebocytes, sebaceous glands, sebum, and lipid signaling | (("Epidermal Growth Factor"[Mesh] OR "Receptor, Epidermal Growth Factor"[Mesh] OR "epidermal growth factor"[tiab] OR EGF[tiab] OR EGFR[tiab]) AND (sebocyte*[tiab] OR "Sebaceous Glands"[Mesh] OR "sebaceous gland"[tiab] OR "sebaceous glands"[tiab] OR sebum[tiab] OR SZ95[tiab] OR lipid*[tiab]) AND (acne[tiab] OR "palmitic acid"[tiab] OR inflammation[tiab] OR inflammatory[tiab])) AND english[lang] AND ("1900/01/01"[dp] : "2026/06/30"[dp]) |
| S4 | Wound healing, re-epithelialization, barrier function, and tissue repair | (("Epidermal Growth Factor"[Mesh] OR "Receptor, Epidermal Growth Factor"[Mesh] OR "epidermal growth factor"[tiab] OR EGF[tiab] OR EGFR[tiab] OR "heparin-binding EGF-like growth factor"[tiab] OR HB-EGF[tiab] OR "transforming growth factor alpha"[tiab] OR epiregulin[tiab]) AND ("Wound Healing"[Mesh] OR "wound healing"[tiab] OR re-epithelialization[tiab] OR reepithelialization[tiab] OR "skin barrier"[tiab] OR "epidermal barrier"[tiab] OR "barrier function"[tiab] OR "tissue repair"[tiab] OR "collagen remodeling"[tiab]) AND (skin[tiab] OR cutaneous[tiab] OR epiderm*[tiab] OR keratinocyte*[tiab])) AND english[lang] AND ("1900/01/01"[dp] : "2026/06/30"[dp]) |
| S5 | Topical formulation, stability, penetration, and delivery systems | (("Epidermal Growth Factor"[Mesh] OR "epidermal growth factor"[tiab] OR EGF[tiab] OR hEGF[tiab] OR rhEGF[tiab]) AND ("Administration, Topical"[Mesh] OR topical[tiab] OR transdermal[tiab] OR cutaneous[tiab] OR dermal[tiab]) AND (formulation*[tiab] OR "drug delivery"[tiab] OR liposome*[tiab] OR transfersome*[tiab] OR emulgel[tiab] OR nanoparticle*[tiab] OR nanocomplex*[tiab] OR microneedle*[tiab] OR iontophoresis[tiab] OR penetration[tiab] OR permeation[tiab] OR bioavailability[tiab] OR stability[tiab] OR conjugate*[tiab] OR mRNA[tiab])) AND english[lang] AND ("1900/01/01"[dp] : "2026/06/30"[dp]) |
| S6 | Fractional laser and post-procedural treatment of atrophic acne scars | (("Acne Vulgaris"[Mesh] OR "acne scar"[tiab] OR "acne scars"[tiab] OR "atrophic acne scar"[tiab] OR "atrophic acne scars"[tiab]) AND ("Epidermal Growth Factor"[Mesh] OR "epidermal growth factor"[tiab] OR EGF[tiab] OR rhEGF[tiab]) AND ("Laser Therapy"[Mesh] OR "fractional carbon dioxide laser"[tiab] OR "fractional CO2 laser"[tiab] OR "carbon dioxide laser"[tiab] OR "CO2 laser"[tiab] OR "laser resurfacing"[tiab] OR postprocedural[tiab] OR "post-procedural"[tiab])) AND english[lang] AND ("1900/01/01"[dp] : "2026/06/30"[dp]) |
| S7 | Contextual acne pathogenesis, guideline-supported therapy, and treatment limitations | (("Acne Vulgaris"[Mesh] OR "acne vulgaris"[tiab]) AND (pathogenesis[tiab] OR sebum[tiab] OR "follicular keratinization"[tiab] OR microbiome[tiab] OR dysbiosis[tiab] OR inflammation[tiab] OR "skin barrier"[tiab] OR lipidomic*[tiab] OR guideline*[tiab] OR retinoid*[tiab] OR "benzoyl peroxide"[tiab] OR antibiotic*[tiab] OR adherence[tiab])) AND english[lang] AND ("1900/01/01"[dp] : "2026/06/30"[dp]) |

**Note:** No automatic human- or animal-study filter was applied because the review intentionally included both clinical and preclinical evidence. Search-result counts should be reported only if they can be recovered from the saved PubMed search history or contemporaneous export; present-day reruns may differ because PubMed indexing is dynamic.
